# Understanding the implementation of telepractice in speech and language services for children and adults using a mixed-methods approach

**DOI:** 10.12688/wellcomeopenres.17622.1

**Published:** 2022-02-09

**Authors:** Varsha Shankar, Vidya Ramkumar, Shuba Kumar

**Affiliations:** 1 Speech, Language and Hearing Sciences, Sri Ramachandra Institute of Higher Education and Research, Chennai, Tamil Nadu, 600116, India; 2 SAMARTH, Chennai, Tamil Nadu, 600004, India

**Keywords:** Implementation, telepractice, speech language pathology, qualitative, scoping review, semi-structured interviews, barriers, facilitators

## Abstract

**Background: **Telepractice emerged as a solution to overcome the challenges of access issues in the delivery of healthcare. Telepractice in speech language pathology (SLP) has existed for nearly a decade yet there is a significant knowledge gap with respect to the factors influencing the implementation of telepractice as a routine or long-term, sustained effort. This mixed-methods study aimed to identify implementation factors that influence the provision of telepractice in SLP services.

**Method: **A mixed-methods study consisting of a scoping review and semi-structured interviews (SSI) was carried out. Articles that described telepractice in SLP were included based on an operational definition of implementation and a set of inclusion criteria.

**Results: **Data was extracted from 11 studies that were mapped to nine projects in telepractice in SLP. The broad focus areas identified included diagnostics and evaluation, therapeutics and comprehensive assessment, management and follow-up care services. Synchronous/ real-time telepractice methods were always used for the provision of diagnostic testing or when providing therapy services using video conferencing. The ‘ *professional-facilitator-patient’* model was used most commonly followed by the ‘ *professional-patient’* model.  Barriers for long-term sustainability included inadequate initial capital investment, lack of reimbursement and payment options, low internet speed and bandwidth, resistance and hesitancy to use telepractice from the patient’s end, lack of organizational policies and uniform regulations. Sustainable source of funding, having a dedicated team of professionals and technicians with clear roles and responsibilities, and inclusion of systematic planning facilitated implementation.

**Conclusion: **In general, telepractice in SLP was not explicitly guided by implementation science or framework. The use of implementation frameworks ensures systematic planning and feasibility assessment to inform the scale-up of implementation. Therefore, it would be worthwhile for program implementers to consider these aspects when exploring telepractice services.

## Introduction

Telepractice-based service delivery is used to enhance health care provision using information and communication technology. It emerged as a solution to overcome the challenges of access issues. Telehealth is playing a crucial role in the delivery of healthcare with the advancement and pervasiveness of technology in daily lives ( Mashima & Doarn, 2008). Telepractice has wide reach and therefore has significant implications in rural and remote locations where opportunities for individuals to obtain specialty consultation or testing are limited. Although it was initially explored in the context of rural/remote regions, its applications in semi-urban and urban regions to overcome scheduling challenges and for issues of convenience have contributed to its appeal. Further, in the context of the COVID-19 pandemic, telepractice based services have added value and it acted as a catalyst in the widespread use of telepractice ( Kollia & Tsiamtsiouris, 2021).

One area where telepractice may be helpful is for treating people with speech language pathology (SLP). Follow-up testing and repeated visits for assessment/therapy/rehabilitation are required as a part of the diagnosis and intervention plan for persons with SLP. The lack of consistent access to these services results in irregular follow-up visits with haphazard schedules, resulting in significant treatment deficits. The availability and accessibility to these services is particularly limited in Low and Middle Income Countries (LMICs), where the majority of the global population live ( Gallego *et al.*, 2017).

Systematic and scoping reviews have reported studies in telepractice in SLP ( Hall *et al*., 2013; Weidner & Lowman, 2020). The purpose of these reviews have primarily been to examine evidence regarding telepractice applications with respect to services delivered for various disorders and details regarding patient-site facilitators. In spite of the benefits of telepractice services, the long-term success of these services is questionable given the lack of data ( van Dyk, 2014). Many telepractice explorations do not last beyond the pilot or experimental phase. There is a significant knowledge gap with respect to use of implementation science and factors influencing the implementation of telepractice service delivery in speech and language services as a routine or long-term, sustained effort.

Implementation science frameworks are useful in contextualizing and systematically planning the implementation of new interventions (like telepractice services) post-experimental or feasibility assessment phases. A holistic implementation approach identifies stakeholder readiness, organizational readiness, and feasibility outcomes to guide implementation. Lack of a systematic approach can lead to implementation errors which in turn result in significant wastage of resources, namely time, human resources and cost. Campbell *et al.* (2020) reported that implementation frameworks were not commonly used to guide the implementation of telepractice services in allied health sciences and there is a failure to employ available robust theoretical models to understand telepractice implementation.

This mixed-methods study aimed to identify implementation factors that influence the provision of telepractice in SLP services. A broad focus was taken for SLP services, including those that cater to all age groups, irrespective of rural or urban regions, implemented on a sustained or routine basis (operationally defined in the methods section). Specific objectives were to 1. identify projects that have an implementation focus and map their geographical distribution through a scoping review 2. understand the focus area, the methods and models of telepractice used in these projects and 3. identify barriers and facilitators that influenced the implementation of telepractice services using a mixed-methods approach consisting of a scoping review and qualitative semi-structured interviews (SSIs).

## Methods

This study received ethical clearance from the Institutional Ethics Committee (Reference Number: CSP/20/NOV/87/191 of Sri Ramachandra Institute of Higher Education and Research (deemed to be University)) 

### Scoping review

***Development of a scoping review protocol.*** A scoping review protocol was developed based on the Preferred Reporting Items for Systematic reviews and Meta-Analyses extension for Scoping Reviews guidelines ( Tricco *et al.*, 2018). The eligibility criteria were based on the Population-Context-Concept framework ( Hannes & Macaitis, 2012). This included search terms, strategies, quality appraisal criteria and extraction methods, as described below.

***Development of search strategy.*** An initial pilot search was carried out using PubMed and Google Scholar. This was used to fine-tune the keywords and the index terms (subject headings) used to identify the articles. The search strategy was iterative as reviewers became more familiar with the evidence base; additional keywords and sources, and potentially useful search terms were discovered and incorporated into the search strategy (available in the Extended data). An initial decision profile (available in the Extended data) was designed to document the selection of articles based on the pilot search.

***Database search.*** PubMed, Cochrane Library, Web of Science, and Scopus electronic databases were searched and grey literature was identified using Google Scholar and ProQuest. The search was conducted between January 2021-April 2021. The articles were screened by title, abstract and then full text against the aforementioned criteria. The citations included in articles were manually screened as a secondary source for articles. Search results were imported into Rayyan QCRI software (Qatar Computing Research Institute, Doha, Qatar) for duplicate removal. This entire process included two reviewers and any disagreements between the two reviewers were discussed until a consensus was reached. The articles were coded based on a coding format developed by the reviewers and a spread-sheet was prepared to extract data from the included studies.

***Inclusion criteria.*** For this study, implementation of telepractice for SLP services was operationally defined as “ *a sustained model of service delivery using telepractice based on prior feasibility and validation studies”*. Any telepractice project implemented for two or more years, providing screening, diagnostic or rehabilitative services to individuals of all age groups with speech and language difficulties across the world, was of interest. Even if the word “implementation” was not used in the articles, words such as “on-going” or the long-term nature of the service implementation described using the number of years was considered. All telepractice modalities, such as the use of video conferencing, web-based, mobile applications and remote computing were included. Literature from January 2010 – April 2021 published in the English language were considered. Quasi-experimental trials, community or field trials, studies describing implementation or feasibility of implementation and fulfilling the operational definition of implementation were included.

***Exclusion criteria.*** Studies involving the comparison of in-person and telepractice measures without long-term implementation were excluded. Also, studies that only reported validation of tests or tools were excluded. Attempts were made to obtain full texts of the articles by writing emails to the corresponding authors or sending full-text requests via ResearchGate. However, articles were excluded when full texts were not available. Studies in languages other than English were also excluded. 

***Analysis*** The Preferred Reporting Items for Systematic Reviews and Meta-Analyses (PRISMA) flow diagram was used to summarize the search results. Data from studies was extracted on: project description, geographical distribution, the focus area of service delivery, method and model of telepractice service delivery. Barriers and facilitators were identified and grouped under five domains *(technical aspects, organizational aspects, patient perspectives, economical aspects, and ethical-legal aspects).* These domains were identified from telemedicine implementation frameworks ( Kidholm *et al.*, 2012; Tanriverdi & Iacono, 1999; van Dyk, 2014). Barriers and facilitators were explicitly reported in some studies based on what they experienced; else suggestions were provided on what was likely to improve the overall implementation. These suggestions were categorized as either barriers or facilitators based on the context of the statement (available in the Extended data). Suggestions, as a quality improvement were considered as facilitators and suggestions as a fundamental requirement that was not available, were considered as barriers.

### Semi-structured interviews (SSI)

***e-Consent.*** Informed e-consent for publication of the participant’s details was obtained from the participants before the semi-structured interview.

***Development of interview guide.*** SSI guides were developed to obtain a detailed understanding of the barriers and facilitators from the perspective of program implementers/ investigators of the projects described in the scoping review. The domains on implementation barriers and facilitators identified in the scoping review served as the framework for the development of the interview guides. The interview guide was initially pilot tested with one project implementer for further refinements and finalization of the guides. A copy of the interview guide can be found in the Extended data.

***Participants.*** The primary and corresponding authors of the studies identified in the scoping review were contacted through email along with a link to an online consent form. Other authors were contacted when responses could not be obtained from the primary and corresponding authors. In a few instances, the investigators were directed to another team member by the primary or corresponding authors themselves. Periodic bi-monthly reminders were provided for scheduling the interview; the consent form was kept open for three months.

***Data collection.*** The SSIs were conducted for five authors who provided consent, via an encrypted online video-conferencing platform at a mutually convenient time. The duration of each interview was approximately 30 to 45 minutes and was carried out in English by the investigators who were trained in conducting qualitative interviews. Interviews were carried out between March 2021 and August 2021.

***Analysis.*** All the audio and video recorded interviews were transcribed verbatim. The transcripts were uploaded into NVivo 12 (QDA Miner Lite is a basic software that is available as a free alternative software). Data were analysed using a hybrid approach of qualitative thematic analysis ( Swain, 2018), which included i) a deductive template of codes and themes derived from our scoping review and ii) a data-driven inductive approach that was carried out following data collection. We then applied the principles of thematic analysis as described by Clarke & Braun (2012) which involved six stages.

In the first stage, we became familiar with our data through repeated readings of the interview transcripts. Next, we coded two transcripts which was done independently by two coders (1st and 2nd author). After independently coding these transcripts we expanded on the existing codebook and added codes inductively derived from the interviews. Any differences in coding were discussed and resolved. The remaining transcripts were coded using this codebook; new issues identified in these interviews relevant to our study were given a new code and inserted into the codebook. We then began to cluster the codes according to similarity and regularity, thereby facilitating the development of categories.

In the third stage, we reviewed these categories and began to search for meaningful patterns in the data relevant to our research questions. Hence, we collated all the coded data relevant to each theme. In the fourth stage, we began to review the themes and to reflect on whether these themes related to our data and were convincing and credible. In the fifth stage, we started to label and define each theme, describing in detail what it signified in the context of our study and the framework derived from the scoping review. Finally, in the 6 ^th^ stage, we brought forth an explanation of our study findings and supported them with quotes that were placed under the respective themes.

## Results

**Figure 1.**
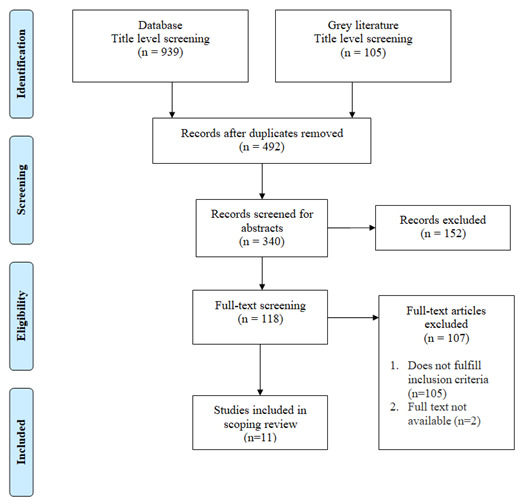
PRISMA flow diagram representing the study’s search process.

This scoping review identified 11 articles that fulfilled the criteria of having an implementation focus for SLP as operationally defined for the purpose of this study ( Figure 1).

### Project mapping

The literature reviewed based on the inclusion criteria of the scoping review has been summarized (available in the Extended data). The data extracted from these studies is tabulated for the purpose of analysis of this scoping review. Table 1a in Extended data includes the study code, study title, authors, countries and focus area. The settings, participants, personnel, and type of services are described in Table 1b in Extended data.

There were several research articles published based on a single large project; to factor this in and make a realistic assessment of implementation focus, project mapping was done. Articles describing common background information in the introduction section of the article, common sites of implementation, broad aims and an overlapping group of investigators were mapped as a single project. Studies that could not be mapped were considered independent projects. Nine projects were identified based on the mapping exercise ( Table 1). Each project was coded for ease of reference as follows: P (project) and serial number. The rest of the findings will be reported using the project codes.

**Table 1.** Summary of project mapping in telepractice in speech language pathology.

Focus area	Country	Research articles	Project code
Diagnostics and evaluation	United States of America	Malandraki *et al*. (2011) Malandraki *et al*. (2012)	P1
	Australia	Burns *et al.* (2019)	P2
Therapeutics	United States of America	Grogan-Johnson (2012) Alvares (2013)	P3
		Boisvert & Hall (2019)	P4
		Short *et al*. (2016)	P5
		Clark *et al*. (2019)	P6
	Australia	Pitt *et al*. (2018)	P7
	Norway	Øra *et al*. (2020)	P8
Comprehensive services	Canada	Haynes & Langevin (2010)	P9

### Geographical distribution of projects

Telepractice in SLP was implemented in five projects from the USA; two in Australia; and one each in Norway and Canada. Projects implemented were predominantly to overcome access barriers for individuals in rural areas and/or remote locations (P2, P3, P5 and P6). Two projects in the US (P1) (P4), one project each in Australia (P7) and Norway (P8) catered to people in both semi-urban and metropolitan areas. None were from LMIC settings.

### Focus area of service delivery

Telepractice based service delivery included; assessment of swallowing disorders for adults and geriatric populations (P1, P2), speech and language therapy services in schools (P3, P4, P5), pediatric feeding therapy (P6), individual and group rehabilitation services for adults with post-stroke aphasia (P7, P8). In one project comprehensive assessment, management and follow-up care services were provided to individuals with stuttering across different age groups using telepractice (P9).

### Method of telepractice

Use of synchronous/real-time, asynchronous/store and forward, or a combination/ hybrid method for providing telepractice services in SLP was identified from the scoping review.

We found that only *synchronous/ real-time methods* were used for telepractice based assessment and therapy services. These include swallowing assessments using real-time video fluoroscopy and clinical swallowing examinations (P1, P2). Therapy services for various speech, swallowing and language disorders were provided real-time using desktop-based video-conferencing software (P3, P4, P5, P6, P7, and P8). Assessment, management and follow up care for individuals with stuttering were also provided using real-time video conferencing (P9).

### Model of telepractice service delivery

Models of service delivery were categorized as: ‘ *professional – facilitator – patient’* where a trained e-helper/telepractice assistant/ support staff supports patient care; ‘ *professional – patient*’ wherein the professional delivers the service directly to the patient without intermediary personnel involved; and ‘ *professional-professional’* wherein a second speech-language pathologist is involved at the patient site.

The ‘ *professional-facilitator-patient’* model was used commonly irrespective of whether services were provided to rural or urban areas. A facilitator/e-helper/paraprofessional supported the service delivery of clinical swallowing assessment (P2) and speech and language therapy services (P3, P4, P5, P9). The ‘ *professional-patient’* model was used to empower the patient’s family member/ caregiver to enable the delivery of therapeutic services for speech and language disorders (P6, P7, P8). The ‘ *professional-professional’* model was reported where an SLP was required for dysphagia assessment, to minimize the risk of aspiration and penetration related incidents (P1).

### Implementation barriers and facilitators

Five of the nine projects reported service delivery to be on-going or routine. The rest, even though long-term, were still only research studies. Feasibility and validation trials within a clinical practice framework were found to be useful in transitioning from the evidence-based research phase to routine implementation (quote 1 in Table 2; all following quotes are also presented in Table 2).

**Table 2.** Reference quotes from the semi-structured interviews.

**Implementation barriers and facilitators**	
1.	“We did a number of trials initially to be able to validate the safety, reliability and validity of the model. That was randomized controlled trials…we actually did those trials within a clinical practice framework … within a hospital environment. They weren't in an academic or a lab based environment… We've also had a really good evidence base to drive our telehealth services and have that quality of care too. And that's been something that's really pushed our service development and the management have really supported that knowing that we have the models that are evidence-based to deliver high quality care. So it's not that we've just come up with it and said, Oh, let's give this a go. We've actually got good rigor in evidence-based practice for that. So that's been another key driver I think, in supporting and expanding our teleservices.” - Project implementer of an on-going telepractice service delivery from Australia
**Patient perspectives**	
2.	“Some people, some families did not want to do it. They wanted to come in, but then we gradually got them to do some sessions face to face and maybe another session or two online. So they do a hybrid approach and people become more used to doing it that way.” - Project implementer from Australia
3.	“We did find that wearing a lapel microphone was important to be able to hear the patient's throat clearing and coughing as if we were sitting with them in an in-person environment.” - Project implementer of an on-going telepractice service delivery from Australia.
4.	“It's a lot more than just a tabletop, desktop, computer, a laptop, or even a phone. We will help support them. We want to make sure technology isn't the barrier. We will always furnish devices for them. If they have a device that's too old to sustain or just can't function effectively, we'll send them a device essentially to lend them while they're participating in the services. We'll send them like a pre-addressed box. They can send it back to us when we're done. And typically when we send folks those, we're sending the iPod touches, which are a little bit cheaper than the iPads or a tablet and they're smaller. And so if anything was to happen to them, we of course have them insured through the university, but it's a low-cost item compared to a $300 tablet or something like that. It's still a cost, but we've had pretty good success rates of getting them back.” - Project implementer of an on-going multidisciplinary telepractice service delivery from USA
5.	“We encourage First Peoples to engage in telepractice. We are very keen to have family members involved and cultural liaison offices who come into the sessions as well to provide support. We've done sessions with interpreters via telehealth, to support people who are from a non-English speaking background. If they're not at the same site, we can link in with them to provide that support. So we try to be very accommodating with whatever the needs are of the person that we're seeing.” - Project implementer of an on-going telepractice service delivery from Australia.
6.	“I think during the past year, many people were fine with it. Towards the end, towards the spring of last year, we had some people who were starting to get antsy that could go back in person.” - Project implementer of an on-going multidisciplinary telepractice service delivery from the USA
**Organizational aspects**	
7.	“It's not a monetary benefit, but an educational benefit. And as we've seen, it's an extremely important part of the curriculum now for speech pathology students, to be able to use technology and to be able to do telepractice, because I think what we're going to see post-COVID is that, people are going to continue to demand to have telepractice. And it's not going to be unusual, it's going to be like normal practice.” - Project implementer from Australia
**Funding**	
8.	“We have funding coming from different sources. One source of funding is through the clinic that our feeding clinic is housed at, which is a program for individuals across their lifespan, who are on the autism spectrum, who have developmental disabilities. What happens in Utah, the state gives this group money, here's X amount of millions of dollars, and you will be the payer and the provider…….. So that's where one source of funding comes from for the clients we see. Funding comes from others - other spaces that we have through my lab, as well as we have a small grant from the Autism Council of Utah.” - Project implementer of an on-going multidisciplinary telepractice service delivery from the USA
9.	“We only meet once per week. So that really helps cut down costs. We probably could meet five days a week if we had the financial capacity, but with what we received now, one day a week allows us to do one to two assessments per week and then treat about five client sessions…… The reason that we were sort of limited now is just that we don't like to practice outside of our capacity, so we're just like one day a week feels good. But if we had, let's say a grant for $50,000, we could fund two graduate students to work 20 hours a week each to do this work. And then that would open up the clinic to more time. We could probably hire on, the fee for service for our speech and language and nutritionist they'll do hourly type work. And then our team typically does more of the intervention type services. So that's where I think we can get a lot more. The problem is finding more funding for, for our graduate students in our training program as part of their practice.” - Project implementer of an on-going multidisciplinary telepractice service delivery from the USA
10.	“We were fortunate here that we have a state-wide telehealth service. So, we actually have a government-funded telehealth network, which has resources and equipment attached to it. So no one really needed to purchase any equipment per se, as part of the implementation, because it's already supplied by the state.” - Project implementer of an on-going telepractice service delivery from Australia
**Organizational administration**	
11.	“An issue is the type of on-site support that our clients have. So depending on the type and severity of the communication disorder, that’s going to – and age of the student, that's going to impact the type of onsite support that's needed. The onsite support is not sufficient. And the student cannot access the services as a result of the lack of onsite support.” - Project implementer of a telepractice service delivery to a school district in the USA
12.	“So we have telehealth coordinators across the different health services who meet with clinicians to try and engage and build and develop their telehealth services. They want people to be able to access services more flexibly, but there's also a reimbursement incentive for the service to do that [telehealth] too. We have administration offices who are very supportive of telehealth and are part of the process. They contact the patients to do test calls prior to the appointment, and they check off their ID, and anything that they would normally do if they were coming in person. So they schedule as part of that too. So it's a new skill set that they've built. And I think that's been a really good thing in terms of getting the admin staff on board to support our services as well. We have a telehealth portfolio leader, which is me in the department. If we have a new platform that comes in or upgrading equipment, or there's new paperwork that comes in about patient appointments, then I get that and I need to then disseminate that out to our admin staff and our clinical staff.” - Project implementer of an on-going telepractice service delivery from Australia
**Equipment and infrastructure**	
13.	“We have a very large state. I think the connection in more of the really rural areas can sometimes be a bit challenging. But it just depends on where they are at and the [internet] traffic and that sort of thing. I know in our own service, we have had sometimes, a variable bandwidth, even in a metropolitan city because of the volume of people that are using it. So I think that's just standard everywhere. If we had increased bandwidth to be able to stabilise connections between sites and a system of consistent high-speed network coverage, then that would take away a lot of challenges.” - Project implementer of an on-going telepractice service delivery from Australia
14.	“So our actual project doesn't really experience that (drop in the internet) because we're providing telepractice to a school district. So the students are in the school building, the school district provides the services and in the state of Ohio, all of our public instruction has a shared infrastructure for the internet. So we have very good internet for that.” - Project implementer of an on-going telepractice service delivery from USA
15.	“From the clinician point of view, I actually have two laptops and both are connected to the same zoom meeting. One is dedicated to audio and video, and the other is dedicated to content. And the reason why I like to do that is sometimes when we share screens in video conferencing, it changes the configuration of the screen. And especially for clinicians who are newer to telepractice, that kind of throws them off a little bit. So having one system that's dedicated just for the audio-video, we have found to be very helpful. And then one system that is just doing the screen sharing for the material works well.” - Project implementer of a telepractice service delivery to a school district in the USA
16.	“The software we use back then …..can now be on an iPad. And we are in the process at the moment of trying to calibrate with the software. It looks as though it's going to work well for us, but we need to do some calibrations with different versions of iPads. The iPads change and then your microphones change. So, it's a matter of trying to get some calibration done now on a number of different versions of the iPad so that we can confidently say that calibration is correct.” - Project implementer from Australia
**Provider’s acceptance of telepractice**	
17.	“When it's thrust upon you like it was during the pandemic - Everybody hated it. Nobody wanted it, but it also was a pandemic. Everything was wrong! And I think it gave telepractice a bit of a black eye, at least when you read the blog posts; some of the speech pathologists are like, I'm not planning for this. I hate this. Why are you making me do this and so I think attitude and how you approach it, it is one very important factor. I think some people are better suited to being a telepractice speech pathologist and it goes to that attitude and that willingness to try, and even if you fail, be a good problem solver and figure it out, I think that does matter.” - Project implementer of an on-going telepractice service delivery from the USA
18.	“Having a good team. So if you have a really strong implementation team of the essential, like stakeholders who are aware of the need for telepractice and are able to just be creative in troubleshooting issues. If you have the people invested and involved, I feel like anything can happen. Like anything positive can happen. We can find ways to access the technology. We can find ways even if it's not synchronous telepractice, if we're saying we're going to have to shift to asynchronous telepractice, having people who value this type of service makes the program. That's very, very important. One can use technology in a host of different ways if you're strategic about it. And if the team really has a strong understanding of the benefits and also the limitations of it. But yeah, I absolutely feel that almost anything is possible if you have the right people involved.” - Project implementer of an on-going telepractice service delivery to a school district in the USA
19.	“Where do you see the barrier to it is more the clinicians because it's a new way of delivering services. And if they have not been prepared properly or trained properly, you will find resistance from clinicians. One of the key barriers is training and preparation - time to have the training have the, to be able to build up their confidence and the knowledge that this will still work for their clients. So they need to see the evidence. And in the training, you have to give them practical skills. The hard part was getting the clinical educators and people to come on board with it and to come into the clinic and learn how to do a session in that way. They were really nervous about it and all that, but, and some were very sceptical that you couldn't do it and that it wouldn't work, but by the time they've done their sessions, of course, they're like they were really into it. The students in fact became very innovative about how they did sessions and how they dealt with the particular clients.” - Project implementer from Australia
**Ethical-Legal aspects**	
20.	“As part of our government-funded standard telehealth network - the telehealth platform that they use allows integration into the facility and also into the home as well. So we actually have a portal that we utilize for patients to use wherever they are on whatever device they're using. So it doesn't really change at all in relation to where the patient is. It still has that support.” - Project implementer of an on-going telepractice service delivery from Australia
21.	“The fact that the government has seen value in this and supported that has been really important. They've set up a reimbursement schedule for telehealth so that there is an incentive for people to use telehealth services. That's paid through the state government. The fact that they've put in a network of resources and equipment for people to use and the telehealth network, both within facilities and in patient's homes.” - Project implementer of an on-going telepractice service delivery from Australia

These sustained implementation efforts included clinical swallowing evaluation conducted within a public health service in Australia (P2), school-based speech and language therapy services provided for children in the USA (P3), and home-based stuttering therapy services provided in Canada (P9).

The results of the scoping review and the thematic analysis of the SSI were combined to report the factors that influenced implementation positively (facilitators) and negatively (barriers). While we began with five domains during the analysis of the scoping review, from the thematic analysis of the SSI, all the data could be identified under the three domains of; *patient/ caregiver related aspects, organizational aspects, and ethical-legal aspects.*

***Patient/Caregiver related aspects.*** Patient/caregivers’ acceptance and perceived benefits and challenges of the services appeared to influence implementation. Perceptions of improved accessibility (less or no travel) to services, reduced wait time, increased consistency and regularity of sessions, reduced cancellations and thereby completion of testing with minimal visits among patients or caregivers were key facilitators to implementation (P2, P6, P7, P8).

Sometimes parents of children undergoing therapy resisted switching over to telepractice services as they perceived it as a hindrance to their child’s performance (P3). When options such as a combination of telepractice and in-person service delivery were provided, it eased their fear and enabled acceptance (refer to quote 2).

Another important facilitator was the availability of devices (e.g. patient/ caregiver laptop, tablets or phones) with necessary adaptations for optimal telepractice services, including swallowing and feeding evaluations (refer to quote 3). In some examples, devices were loaned to the patients for the telepractice sessions so they were not required to procure any new equipment to engage in the service (refer to quote 4).

Patient preparation and orientation to the processes involved in using telepractice entailed intensive counseling sessions, handing out information resources and providing them with assistance for technical issues (P7, P8), all of which were found to be helpful. This smooth transition was reported to create ease and better adoption of telepractice. Attention to socio-cultural aspects was reported to enhance acceptance of telepractice and enable accessibility (refer to quote 5).

While COVID-19 acted as a catalyst in the widespread use of telepractice, after a point when patients/ caregivers had reached saturation, they looked forward to reverting to traditional in-person services (refer to quote 6).

***Organizational aspects.*** Organizations tend to provide economic, human resource, and infrastructural support for sustained implementation of telepractice, when such services in turn result in monetary profits or non-monetary benefits (e.g. student training opportunities). Some institutions supported telepractice ventures due to the educational benefit associated with it for the students enrolled in their graduate programs (refer to quote 7).

*Funding:* Predominantly, services were funded through research grants obtained from charitable donations and governmental organizations (P1, P3, P9) (refer to quote 8). When services were not initiated through research grants, then the higher initial capital investment required to support telepractice was a challenge (P1, P2). Also, obtaining funding to hire more personnel to support the expansion of telepractice services was another key challenge (refer to quote 9). Prior assessment of the availability of internal resources (infrastructure, funding, equipment, human resources) was beneficial in optimizing implementation (P3). Program implementers reported that using and optimizing existing equipment proved to be facilitatory as it minimized any additional costs (refer to quote 10).

*Organizational administration:* Inconsistencies in billing codes, services covered by insurance companies, and a variety of administrative steps, negatively impacted telepractice service delivery (P6). Reimbursements from insurance companies as well as non-availability of digital payment portals were also reported to be barriers (P3). Self-sustaining, billable implementations of telepractice in SLP were very few (P6, P9).

Lack of onsite support affected service delivery (P1, P3) (refer to quote 11). A dedicated administrative and technical support team supported successful implementation (P5). Telepractice was more successful when roles and responsibilities of the e-helper/facilitator were clearly described. Incentivizing providers for telepractice adoption was considered a useful measure to promote provider satisfaction that could enhance the sustainability of such services (refer to quote 12).

*Equipment and infrastructure:* Drops in internet speeds were reported especially during peak usage times (P9) affecting service delivery. Poor internet bandwidth affected image quality or intermittency in audio and video transmission. Variable bandwidth made it challenging for off-site clinicians to perform necessary assessments or provide therapy (P1, P5, P7 and P8) (refer to quote 13).

The use of virtual private networks (VPN) and Internet Protocol Wide Area Network (IPWAN) facilitated seamless delivery of telepractice services due to the increased bandwidth availability and more reliable and stable internet connectivity (P5) (refer to quote 14). The use of high quality dedicated video-conferencing equipment was recommended for quality enhancement in the transmission of video and audio (P5). Video-conferencing systems with closed captioning were an advantage in telepractice services (refer to quote 15).

Periodic upgradation of software and technology interfaces with constant advancements were incorporated (refer to quote 16)

*Provider’s acceptance of telepractice:* Adoption of telepractice seemed to evoke negative feelings and apprehensions initially and this was more evident during the start of the COVID-19 pandemic (refer to quote 17). Providers felt inadequately trained and sceptical to use technology, and were apprehensive of these changes/new methods of providing services.

Training additional personnel as telepractice assistants helped bring about a smooth transition in the event any of the assistants took a day off or else quit the job. Systematic training using a variety of materials and methods were considered useful. Tele-supervision helped facilitate strong relationships between the personnel. In addition to clinical strategies, an understanding of the technology used, the ability to troubleshoot, and basic orientation of security measures had to be dealt with during training sessions (P4). Team building, coordination and communication between all providers involved in service delivery resulted in reliable patient outcomes (P3) (refer quote 18).

Upon seeing positive outcomes in patients, providers felt satisfied (P4) (refer to quote 19). Some providers also began to appreciate their ability to adapt to telepractice service delivery and serve as key facilitators in sustaining services (P3, P4).

### Ethical-Legal aspects

In some countries lack of national regulations, or differential regulations in each state or province posed restrictions on telepractice services (P6, P9). Having the technical infrastructure for secured transmission of patient data was an essential requirement and helped in the implementation of telepractice services (P1) (refer to quote 20). Interview participants also reported that when data security, privacy and confidentiality were honoured, patients felt secure (refer to quote 21).

## Discussion

The current mixed methods study suggests that telepractice is considered as an alternative/ supplementary service delivery to primarily cater to remote rural areas or when there are other practicality issues in accessing services in-person. This may be due to the regulatory guidelines existing in various countries or an overall preference for in-person services for health-related consultations ( Cason & Cohn, 2014; Swanepoel *et al.*, 2010). Though, there is a change in this situation post COVID-19 pandemic, yet it appears that there is an ‘online fatigue’ and the sudden increase of telepractice services during COVID-19 may be a temporary phenomenon ( Jaffar & Ali, 2021).

Studies included in the scoping review provided adequate details of the type of disorder, age of beneficiaries and the methods and models used to provide services using telepractice in specific delivery settings. This information is likely to be useful to program planners across countries. However, impact evaluations were limited with only one study reporting cost-outcomes, yet these findings are not generalizable to other contexts.

Although, we came across studies that reported telepractice service delivery in SLP in LMICs, they did not fit the inclusion criteria as they were predominantly pilot studies or validity trials (e.g. Chan *et al*., 2021; Pamplona & Ysunza, 2020; Raman *et al.*, 2019). Telepractice service delivery was not reported in the context of long-term implementation. Considering the higher disparity in demand versus capacity the need for telepractice in LMICs, addressing the research gap in outcomes and impact evaluations of sustained implementation in LMICs is pertinent ( Khoza-Shangase *et al.*, 2021).

In telepractice applications in SLP, only *synchronous/real-time methods* were used for both assessment and therapy. There was a preference for this method as real-time judgment of the SLP was considered critical. Telepractice services were provided to adults in hospitals, or in community clinics or even homes, and for children it was provided in their school or home setting.

The ‘ *professional-facilitator-patient’* model was used commonly irrespective of whether services were provided to rural or urban areas. Real-time video conferencing eliminated the need for the presence of a remote professional or support staff in the *‘professional-patient’* model. In these cases, the parent/caregiver served as a facilitator. Some level of patient preparation was involved in this model. Only when simple technologies were required or when the assessment did not require high level of skills, was it possible to provide telepractice services at homes. The ‘ *professional-professional'* model was generally less used, since predominantly telepractice services were implemented to overcome limitations in professional resources, especially in the rural context.

In general, implementation frameworks were not used to guide the planning, provision or outcome evaluations of telepractice services in SLP. While only one project used an implementation framework - the Consolidated Framework for Advancing Implementation Research ( Damschroder *et al.*, 2009) - to guide their work (P2), others ‘learnt-on-the-go’ by making adaptations along the way.

Organisational aspects had substantial influence on implementation. Funding, administrative and infrastructure support were key elements that emerged as a part of organisational support from this study. We found that telepractice services that received public-funding reported better sustainability. Such public-funding support is possible when policy makers consider telepractice as one essential arm of service delivery. Availability of critical infrastructure such as internet also depends on political will of administrators. For example; with COVID-19, efforts were made to improve internet access and speed, yet, not much changed in rural/remote areas where telepractice needs were the most ( Lai & Widmar, 2021).

Telepractice services in SLP were implemented with simple video-conferencing technologies that could be directly delivered to the patient in their homes ( Boisvert *et al.,* 2012). Only when services required instrumentation, complex intervention, or skilled assistant, a larger scale of implementation was reported. Even when transitions had to be made in the interfacing software or hardware due to technological advancements, such adaptations were made as and when required.

Finally, policies, guidelines, laws are required to ensure that services are provided within ethical and legal boundaries. Wherever national or state laws are not available, individual organizations may evolve their practice guidelines to protect the interest of patients, providers ( Cason & Brannon, 2011) and program implementers thereby enabling them to take confident steps towards telepractice implementation.

The strength of the current study lies in the unique method of using a mixed-methods approach (combining a scoping review and semi-structured interviews) to get a deeper understanding of the barriers and facilitators influencing the provision of telepractice services in SLP. Considering the small number of studies with implementation focus, quality appraisal was not carried out, yet this is a limitation of the study. Another limitation is with respect to the restricted context of the views that were obtained from the qualitative SSIs, as all participants belonged to only two countries.

## Conclusion

In general, telepractice in SLP was not explicitly guided by implementation science or framework. Only one project used an implementation framework to guide their work. Factors such as initial capital investment, lack of reimbursement and payment options, reduced internet speed and bandwidth, resistance and hesitancy to use telepractice from the patient’s end, lack of organizational policies and uniform regulations were found to be barriers for long-term sustainability. Sustainable source of funding, having a dedicated team of professionals and technicians with clear roles and responsibilities, and inclusion of systematic planning facilitated implementation.

The findings from this study can guide the planning of future telepractice based services in SLP. Telepractice implementation research and reporting in the LMIC context will be valuable considering the demand for such services. And finally, all program planners may consider the use of implementation frameworks for robust and systematic planning, feasibility assessment, adaptations before full-scale implementation.

## Data availability

### Underlying data

The data underlying this study consists of transcripts of the qualitative interviews that identify the participants and cannot be sufficiently de-identified. To ensure confidentiality and data protection, we have only included relevant excerpts from the interviews with ‘no objections’ from the participants. Any specific qualitative data set underlying this study can be made available on request to the corresponding author. We will only provide the transcripts to researchers/those that provide a proposal of how the data will be used by them. The corresponding author will ensure to remove identifiers from the particular data set and obtain ‘no objections’ from the participants before sharing the same.

### Extended data

Open Science Framework: Understanding the implementation of telepractice in speech and language services for children and adults using a mixed-methods approach

https://doi.org/10.17605/OSF.IO/C7GRX ( Shankar *et al*., 2022).

This project contains the following extended data:

- Search terms used for the article selection
- Initial Decision Profile
- Barriers and facilitators reported in SLP projects
- A copy of the questionnaire
- Table 1a and 1b: Summary of articles identified in telepractice in speech language pathology

Data are available under the terms of the Creative Commons Zero "No rights reserved" data waiver (CC0 1.0 Public domain dedication).

## References

[ref-1] Alvares R : Working With Facilitators to Provide School-Based Speech and Language Intervention via Telepractice. *Perspectives on Telepractice.* 2013;3(2):44–48. 10.1044/teles3.2.44

[ref-2] Boisvert M Hall N Andrianopoulos M : The Multi-faceted Implementation of Telepractice to Service Individuals with Autism. *Int J Telerehabil.* 2012;4(2):11–24. 10.5195/ijt.2012.6104 25945200 PMC4296824

[ref-3] Boisvert MK Hall N : Telepractice for School-Based Speech and Language Services: A Workload Management Strategy. *Perspectives of the ASHA Special Interest Groups.* 2019;4(1):211–216. 10.1044/2018_PERS-SIG18-2018-0004

[ref-4] Burns CL Ward EC Gray A : Implementation of speech pathology telepractice services for clinical swallowing assessment: An evaluation of service outcomes, costs and consumer satisfaction. *J Telemed Telecare.* 2019;25(9):545–551. 10.1177/1357633X19873248 31631757

[ref-5] Campbell J Theodoros D Hartley N : Implementation factors are neglected in research investigating telehealth delivery of allied health services to rural children: A scoping review. *J Telemed Telecare.* 2020;26(10):590–606. 10.1177/1357633X19856472 31216211

[ref-6] Cason J Brannon JA : Telehealth Regulatory and Legal Considerations: Frequently Asked Questions. *Int J Telerehabil.* 2011;3(2):15–18. 10.5195/ijt.2011.6077 25945185 PMC4296804

[ref-7] Cason J Cohn ER : Telepractice: An Overview and Best Practices. *Perspectives on Augmentative and Alternative Communication.* 2014;23(1):4–17. 10.1044/aac23.1.4

[ref-8] Chan MY Chu SY Ahmad K : Voice therapy for Parkinson’s disease via smartphone videoconference in Malaysia: A preliminary study. *J Telemed Telecare.* 2021;27(3):174–182. 10.1177/1357633X19870913 31431134

[ref-9] Clark RR Fischer AJ Lehman EL : Developing and Implementing a Telehealth Enhanced Interdisciplinary Pediatric Feeding Disorders Clinic: a Program Description and Evaluation. *J Dev Phys Disabil.* 2019;31(2):171–188. 10.1007/s10882-018-9652-7

[ref-10] Clarke V Braun V : Teaching thematic analysis: Overcoming challenges and developing strategies for effective learning. *The SAGE Encyclopedia of Qualitative Research Methods.* 2012. 10.4135/9781412963909.n451

[ref-11] Damschroder LJ Aron DC Keith RE : Fostering implementation of health services research findings into practice: A consolidated framework for advancing implementation science. *Implement Sci.* 2009;4(1):50. 10.1186/1748-5908-4-50 19664226 PMC2736161

[ref-12] Gallego G Dew A Lincoln M : Access to therapy services for people with disability in rural Australia: a carers' perspective. *Health Soc Care Community.* 2017;25(3):1000–1010. 10.1111/hsc.12399 27753195

[ref-13] Grogan-Johnson S : Take the Tele-Plunge at Your School An Ohio group shares five key steps to setting up remote speech- language treatment in schools. 2012. 10.1044/leader.FTR1.17122012.10

[ref-14] Hall N Boisvert M Steele R : Telepractice in the Assessment and Treatment of Individuals with Aphasia: A Systematic Review. *Int J Telerehabil.* 2013;5(1):27–38. 10.5195/ijt.2013.6119 25945211 PMC4296832

[ref-15] Hannes K Macaitis K : A move to more systematic and transparent approaches in qualitative evidence synthesis: update on a review of published papers. *Qual Res.* 2012;12(4):402–442. 10.1177/1468794111432992

[ref-16] Haynes E Langevin M : Telepractice at the Institute for Stuttering Treatment and Research (ISTAR). *Stuttering Awareness Day Online Conference for.* 2010. Reference Source

[ref-17] Jaffar R Ali AZ : Examining Ease and Challenges in Tele-Assessment of Children Using Slosson Intelligence Test. 2021;36(4):555–570. Reference Source

[ref-18] Khoza-Shangase K Moroe N Neille J : Speech-Language Pathology and Audiology in South Africa: Clinical Training and Service in the Era of COVID-19. *Int J Telerehabil.* 2021;13(1):e6376. 10.5195/ijt.2021.6376 34345349 PMC8287713

[ref-19] Kidholm K Ekeland AG Jensen LK : A model for assessment of telemedicine applications: Mast. *Int J Technol Assess Health Care.* 2012;28(1):44–51. 10.1017/S0266462311000638 22617736

[ref-20] Kollia B Tsiamtsiouris J : Influence of the COVID-19 pandemic on telepractice in speech-language pathology. *J Prev Interv Community.* 2021;49(2):152–162. 10.1080/10852352.2021.1908210 33843493

[ref-21] Lai J Widmar NO : Revisiting the Digital Divide in the COVID-19 Era. *Appl Econ Perspect Policy.* 2021;43(1):458–464. 10.1002/aepp.13104 33230409 PMC7675734

[ref-22] Malandraki G McCullough G He X : Teledynamic Evaluation of Oropharyngeal Swallowing. *NIH Public Access.* 2011;23(1):1–7. 10.1044/1092-4388(2011/10-0284) PMC4165336 22052284

[ref-23] Malandraki G McCullough G Perlman A : Dysphagia Assessment at a Distance. *The ASHA Leader.* 2012;17(5):26–28. 10.1044/leader.FTR3.17052012.26

[ref-24] Mashima MA Doarn CR : Overview of telehealth activities in speech-language pathology. *Telemed J E Health.* 2008;14(10):1101–17. 10.1089/tmj.2008.0080 19119834

[ref-25] Øra HP Kirmess M Brady MC : Technical Features, Feasibility, and Acceptability of Augmented Telerehabilitation in Post-stroke Aphasia-Experiences From a Randomized Controlled Trial. *Front Neurol.* 2020;11:671. 10.3389/fneur.2020.00671 32849176 PMC7411384

[ref-26] Pamplona MdC Ysunza PA : Speech pathology telepractice for children with cleft palate in the times of COVID-19 pandemic. *Int J Pediatr Otorhinolaryngol.* 2020;138:110318. 10.1016/j.ijporl.2020.110318 32871515 PMC7428427

[ref-27] Pitt R Hill AJ Theodoros D : “I definitely think it’s a feasible and worthwhile option”: perspectives of speech-language pathologists providing online aphasia group therapy. *Aphasiology.* 2018;32(9):1031–1053. 10.1080/02687038.2018.1482403

[ref-28] Raman N Nagarajan R Venkatesh L : School-based language screening among primary school children using telepractice: A feasibility study from India. *Int J Speech Lang Pathol.* 2019;21(4):425–434. 10.1080/17549507.2018.1493142 30175626

[ref-29] Shankar V Ramkumar V Kumar S : Understanding the implementation of telepractice in speech and language services for children and adults using a mixed-methods approach. 2022. 10.17605/OSF.IO/C7GRX PMC9490278 36158869

[ref-30] Short L Rea T Houston B : Positive Outcomes for Speech Telepractice as Evidence for Reimbursement Policy Change. *Perspectives of the ASHA Special Interest Groups.* 2016;1(18):3–11. 10.1044/persp1.sig18.3 30221200

[ref-36] Swain J : A Hybrid Approach to Thematic Analysis in Qualitative Research: Using a Practical Example.2018. 10.4135/9781526435477

[ref-31] Swanepoel DW Clark JL Koekemoer D : Telehealth in audiology: The need and potential to reach underserved communities. *Int J Audiol.* 2010;49(3):195–202. 10.3109/14992020903470783 20151929

[ref-32] Tanriverdi H Iacono CS : Diffusion of telemedicine: A knowledge barrier perspective. *Telemed J.* 1999;5(3):223–244. 10.1089/107830299311989 10908437

[ref-33] Tricco AC Lillie E Zarin W : PRISMA extension for scoping reviews (PRISMA-ScR): Checklist and explanation. *Ann Intern Med.* 2018;169(7):467–473. 10.7326/M18-0850 30178033

[ref-34] van Dyk L : A review of telehealth service implementation frameworks. *Int J Environ Res Public Health.* 2014;11(2):1279–1298. 10.3390/ijerph110201279 24464237 PMC3945538

[ref-35] Weidner K Lowman J : Telepractice for Adult Speech-Language Pathology Services: A Systematic Review. *Perspectives of the ASHA Special Interest Groups.* 2020;5(1):326–338. 10.1044/2019_persp-19-00146

