# Supplementary material for: Understanding the implementation of telepractice in speech and language services using a mixed-methods approach
Source: Wellcome Open Res. 2022 May 26;7:46. Originally published 2022 Feb 9. [Version 2] doi: 10.12688/wellcomeopenres.17622.2 (PMC9490278; doi:10.12688/wellcomeopenres.17622.2)
Supplement: Peer review history

# Peer review: Reviewer response for version 2

**DOI:** 10.21956/wellcomeopenres.19853.r52218

**Published:** 2022-09-20

**Authors:** Mark Krumm

**Affiliations:** 1 Department of Hearing Sciences, Kent State University, Kent, OH, USA

I very much enjoyed reading "Understanding the implementation of telepractice in speech and language services using a mixed-methods approach".

 This article provides an important contribution to telepractice patterns and issues provided by speech and language pathologists today. This article details common models and barriers of telepractice and attempts to determine if a framework is consistently used in successful long-term telepractice programs. In turn, the authors identify practices building toward a best practice framework that others may follow when considering the provision of telepractice. The authors make a clear case that a guiding framework is not often used to achieve best practice or to develop strategies for overcoming barriers. Literature-based research is provided by the authors in this paper which are key to a successful telepractice service regarding equipment, funding, personnel, client needs, and planning. The authors believe a data-based framework is indicated for today's practitioners, and given the evidence they describe in this article, it is hard to argue against them. 

 It may seem difficult to realize that after all of these years telepractice in speech and language pathology still is struggling with issues identified in this article. I feel this article also correctly identifies issues that are critical, but perhaps not considered seriously or in-depth, such as service sustainability, legal practices, technology, and personnel/client resistance to telepractice. I liked this article because the barriers and best practices associated with telepractice are rarely addressed holistically, and therefore, not easily generalized to other telepractice environments. This article provides that holistic view that can be used to guide individuals attempting to develop a telepractice program. Because of the holistic nature of this paper, it is also a substantial contribution to the literature for clinicians and researchers alike. Yes, some of the barriers have been known for a long time (e.g. telepractice projects not sustained beyond grant funding), but these problems still persist today and should be recognized before implementing a telepractice program.

 In summary, I feel this article provides an excellent article for both best practices and the need to plan strategically for barriers in order to ensure valuable and sustained telepractice services. I think there is much to be learned from this article which extends beyond speech and language pathology telepractice into other health care professions, including audiology. I think this is a must-read paper for clinicians and investigators alike contemplating a telepractice program startup or enhancement.

 In the section below are some edits to the text that I think might be helpful:

**In the results of the abstract, the authors wrote**:

 "resistance and hesitancy to use telepractice from the patient’s end,"

- **Change recommended:** patient resistance and hesitancy to use telepractice.

**In the second paragraph of the Introduction, the authors wrote:**

 "and a AUD 40.05 savings per patient per referral."

- **Change recommended:** Provide the term or phrase for AUD and place AUD in parenthesis following the term or phrase.

**In the third paragraph of page 10 of 20 the authors wrote:**

 "Periodic upgradation of software and technology interfaces with constant advancements were incorporated"

- **Change recommended**: Substitute upgradation with “upgrades”.

**On page 10, first paragraph under conclusions, the authors wrote:**

 "The current mixed methods study suggests that telepractice is considered as an alternative/ supplementary service delivery to primarily cater to remote rural areas or when there are other practicality issues in accessing services in-person."

- **Change recommended: **Delete the phrase: “to primarily cater to”, add the phrase: "primarily employed for applications in”.
- **Change recommended:** Delete the phrase: ”practicality issues”, add the phrase: “barriers”.

**On page 10, first paragraph under conclusions section, the authors wrote:**

 "Though, there is a change in this situation post COVID-19 pandemic, **yet **it appears that there is an ‘online fatigue’ and the sudden increase of telepractice services during COVID-19 may be a temporary phenomenon (Jaffar & Ali, 2021)"

- **Change recommended: **Delete: “yet” in the paragraph above, no word insertion in lieu of “yet”.

**On page 10, second paragraph under conclusions section, the authors wrote:**

 "However, impact evaluations were limited with only one study reporting cost-outcomes, yet these findings are not generalizable to other contexts"

- **Change recommended:** Remove comma after “cost-outcomes” and insert a “.” (period) instead to make this sentence two sentences.
- **Change recommended: **Remove “yet” and insert “Unfortunately,”                    

**On page 10, last paragraph on the page, the authors wrote:**

 "Only when simple technologies were required or when the assessment did not require high level of skills, was it possible to provide teleprac[1]tice services at home"

- **Change recommended:** Home telepractice services were conducted at home, but only when basic technology configurations were required, or when the assessment protocol did not require a high degree of clinician telepractice skill levels.
  (Note: The reviewer is with the above sentence that it is the clinician who did not need a high skill level in telepractice, but did this statement also mean that the client did not need to have high skill levels with telepractice technology in addition to the clinician?)

**On page 10, last paragraph on the page, the authors wrote:**

 "The ‘professional-professional’ model was generally less used, since predominantly telepractice serv[1]ices were implemented to overcome limitations in professional resources, especially in the rural context"

- **Change recommended:** Generally, the ‘professional-professional’ model was the least frequently model. This is not surprising given the key rationale for implementing telepractice services was to overcome limitations in professional resources (especially in rural settings).

**On page 11, second paragraph,** **the authors wrote:**

 "We found that telepractice services that received public-funding reported better sustainability"

- **Change recommended:** Delete the second “that received” in the sentence and replace it with “which was supported by”.

**On page 11, paragraph 3, the authors wrote:**

 Only when services required instrumentation, complex intervention, or skilled assistant, a larger scale of implementation was reported. Even when transitions had to be made in the interfacing software or hardware due to technological advancements, such adaptations were made as and when required

- **Change recommended:** A larger scale of implementation was reported only when services required instrumentation, complex intervention, or skilled assistant became necessary.
- **I think you also eliminate the following sentence as it seems redundant: **"Even when transitions had to be made in the interfacing software or hardware due to technological advancements, such adaptations were made as and when required"

**On page 11, paragraph 4, the authors wrote:**

 "Considering the small number of studies with implementation focus, quality appraisal was not carried out, yet this is a limitation of the study."

- **Change recommended:** Eliminate the comma after “out” and eliminate the word “yet” and insert “and therefore this issue” before “is a limitation of the study”.

Is the work clearly and accurately presented and does it cite the current literature?

Yes

If applicable, is the statistical analysis and its interpretation appropriate?

Yes

Are all the source data underlying the results available to ensure full reproducibility?

Yes

Is the study design appropriate and is the work technically sound?

Yes

Are the conclusions drawn adequately supported by the results?

Partly

Are sufficient details of methods and analysis provided to allow replication by others?

Yes

Reviewer Expertise:

Telehealth in the speech and hearing sciences.

I confirm that I have read this submission and believe that I have an appropriate level of expertise to confirm that it is of an acceptable scientific standard.

# Peer review: Reviewer response for version 1

**DOI:** 10.21956/wellcomeopenres.19494.r49293

**Published:** 2022-04-08

**Authors:** K. Ganapathy

**Affiliations:** 1 Apollo Telemedicine Networking Foundation, Chennai, Tamil Nadu, India

The authors are to be complimented for doing an in-depth analysis of a subject not usually given the importance it deserves, namely studying factors influencing the **actual *implementation* of telepractice. **The authors being domain experts in speech and language pathology (SLP) have rightfully confined themselves to the study of the implementation of technology-enabled remote SLP services. An analysis of scoping reviews and semi-structured interviews has been meticulously carried out. Deploying standard accepted procedures (inclusion, exclusion, PRISMA criteria, data collection, analysis), a thorough scientific study of 11 shortlisted publications has been done.  Inadequate initial capital investment, lack of reimbursement and payment options, low internet speed, resistance and hesitancy to use telepractice from the patient’s end, lack of organizational policies, and uniform regulations were the identified barriers to implementation. A sustainable source of funding, having a dedicated team of professionals and technicians with clear roles and responsibilities, and inclusion of systematic planning facilitated implementation. The authors have stressed that in the papers studied telepractice implementation in SLP was not guided by Management Science. The lack of a systematic approach led to implementation errors. The authors have quoted verbatim responses from those interviewed. This approach gives the reader a clearer perspective than using routine mundane emotionless terminology, a hallmark of scientific communication! The reader now has actual views of those who have rolled up their sleeves and made their hands dirty! Areas covered in this review paper include implementation barriers and facilitators, patient perspectives, organisational aspects, funding, equipment and infrastructure, providers’ acceptance of telepractice, and ethical and legal aspects. The authors discuss the differences between the *professional-facilitator-patient*, *professional-patient,* and *professional-professional* groups.

 Discussion regarding sustainability, more information on the business model, cost-effectiveness, some data on the market for telepractice, and some info on the acute shortage of trained audiologists and speech experts would be useful. Some statistics from a few countries in different income brackets ( https://datahelpdesk.worldbank.org/knowledgebase/articles/906519-world-bank-country-and-lending-groups) would convince the reader that this gap in SLP management is unlikely to be redressed without virtually extending the reach of the limited domain experts available. While the primary intent of this study is to summarise the current world literature (choosing articles fulfilling very rigid criteria), it perhaps may not be irrelevant to include interviews with others deploying telepractice in SLP in India, including the authors themselves (assuming that the quotes could be subsumed under Personal Communication). This addendum may add to the perspective. The low availability and accessibility of SLP services in low and middle-income countries (LMICs) can also be discussed.

 Authors have pointed out that none of the papers studied in deploying telepractice in SLP were from LMICs. It needs to be pointed out that even if such projects were being executed in LMICs, the priority for publishing would be low. Publishing is a time-consuming, labor incentive process, particularly in a non-academic milieu. Though implied, the authors need to reiterate that their conclusions are entirely a synopsis of the 11 studies shortlisted. Five projects from the USA, two from Australia, one each from Norway and Canada do not represent the world. Even guidelines published for the implementation of telehealth, whether it be for SLP or any other clinical domain, are not fixed. It could vary based on changing technology, perceptions, behavioural modifications, reimbursement policies, and a myriad other factors. Success lies in identifying these variables, giving proper weightage to each of them, and being willing to take a relook periodically. One or two paragraphs highlighting the limitations of the methodology used in this study would add value to the paper. Reliable information available in the public domain, though not in indexed journals, could also be mentioned

 Telehealth is a major innovation at the technological, social, and cultural levels. Telehealth projects are complex, innovative, and continually evolving. All responses cannot be anticipated. Telehealth generally has a poor record of long-term sustenance. Few organisations have succeeded in reaching large-scale, enterprise-wide adoption. The reviewer, though not an SLP expert, has been actively involved in the management of scores of small and mega telehealth projects over the last 22 years ( https://www.youtube.com/watch?v=Wc__mhGDpEE ^1^) ^2^ and therefore **now** clearly understands the importance of deploying Management Science. Operational, technical, and clinical challenges in virtually reaching the unreached are dependent on geography, time, and socio-economic environment. Principles of Management Science need to be customised, tailor-made, and adapted as and when required. Publications need to stress organisational, regulatory, and cultural issues *vis a vis* its deployment in initiating, sustaining, and making viable telehealth activities. The WHO framework for the implementation of Telemedicine Services gives some guidelines ^3^. ****Self-sustaining telehealth projects need to be revenue-generating. The reviewer while initiating telehealth projects across India and a few overseas had to fall back upon discussions with a passionate, street-smart team. Interestingly, it was realised that lessons learnt in the field actually complied with the dicta of management gurus enunciated years later! A detailed need assessment study was the first step. Risks in program implementation, challenges in providing specialists, the necessity for training, retraining, learning, relearning, and unlearning were emphasised. Identifying champions in the community and the team was critical. Future-ready access to cost-effective, need-based, appropriate technology including a robust perspective of telemedicine quality must be considered. Measurable, reproducible, objective parameters to quantify success needs to be defined. Barriers need to be foreseen and addressed. Legal, regulatory, and security issues have to be complied with. The cultural transformation of the beneficiary, health care provider, and the local society is critical. Political will and support go a long way in implementing a telehealth project, particularly when it involves rehabilitation among the young as in SLP projects, where the benefits are not immediate or dramatic. Urban specialists need to be sensitized to community interactions while deploying cutting-edge technology. Programme Managers need to be transparent, accountable, responsible, and open to external third-party audits. Quality of service through feedback needs to be measured. Key Performance Indicators need to be defined. Channels of communication and grievance redressal mechanisms are required, as are escalation and evaluation matrices.  

 Technology acceptance is always a concern. A detailed customised Standard Operating Procedure is necessary. Evidence of cost-effectiveness is necessary for widespread telemedicine adoption ^4^^,^^5^^,^^6^, ****ensuring funding and sustainability are necessary ^7^. Donors, governments, cross-subsidy, for-profit companies, and rarely venture capitalists have been sources of funding. Successful telehealth applications are often run by local telemedicine champions with *ad hoc *funding. Emphasizing personal user advantage and incentivization is as important as advocating general societal advantages.

 Barriers to the adoption of telemedicine include difficulties in technology integration, interoperability, standar­dization, security, time constraints, and financing. Technology can be provided, but health professionals need to use it. Their percep­tions, legal issues, technical difficulties, time, convenience, and cost are critical. Hurdles relate to reimbursement, policies governing telecommunication and information technologies, development, and licensure. Acceptance by clinicians and beneficiaries, demonstrable savings, improved access to healthcare, reduced effort, travel time, and adoption into daily practice leads to successful teleme­dicine applications. Adoption of telemedicine, patient satisfaction, and doctor-patient interactions depend considerably on utilisation of Human Management principles. The latter is intuitive. If patients perceive that telemedicine will save time/money and increase comfort, they may adopt telemedicine. Perceptions before the teleconsult shape decision to (a) sample telemedicine services and (b) use telehealth services regularly. The greater the perceived advantage and perceived compatibility, the greater the intent. To ensure effective operation, skillful planning and implementation are as essential as system design, software functionality, and technical prowess. Optimization of work processes is important. Operations need to be re-invented to use newly available information. Identifying significant pain points results in new innovative solutions.

 There are several mega ongoing telehealth projects in India that have stood the test of time, are self-sustaining, and are revenue-generating. Most of these are from non-academic institutions with little or no resources for publishing. The “tricks of the trade” may also be considered confidential and may not be made available in the public domain and hence would not come under the purview of a rigorous scoping review ( https://www.youtube.com/watch?v=yxeuCWBOExM ^8^). Interestingly, the authors of this publication are from India. With 63 million having hearing impairment ^9^, 14 to 20% of pre-school children having delayed or disordered speech and language, if any country needs to understand the dos and don'ts in the implementation of telepractice in speech and language services, it is India. Hopefully, the authors' stringent analysis will help commence the much-needed awareness. Biraj Bhattarai ^10^ in a study of 118 participants from India, found that only 16% were properly trained to provide teleservices. Mohan *et al.* ^11^ analysing responses of 205 SLP practitioners, noted that only 12.19% used telepractice. Considering that in 2015, as per the Indian Speech and Hearing Association, there were only 2500 SLP practitioners, telepractice appears to be the only way to virtually extend *their *reach.

**Reviewer’s additional comments**

**MAJOR:**

- **Is the work clearly and accurately presented and does it cite the current literature?**

The work is clearly and accurately presented. Due to the stringent measures adopted and rightly so, the literature cited is ultra-focused based on search strategy, inclusion, exclusion criteria, PRISMA method, etc. However for the reader to get the “Big Picture”, references to the literature dealing with the **business **of telehealth, Management Science in telehealth, and factors directly involved in the deployment of telehealth can be added.

- **Is the study design appropriate and is the work technically sound?**

Yes.

- **Are sufficient details of methods and analysis provided to allow replication by others?**

Yes, details have been provided though numbers are so small and this being a recent topic may not be much scope for immediate replication. The authors define implementation as a sustained model of service. It is desirable that the reader understands the duration of the viability of each of the projects analysed (minimum, maximum, info not available, etc with reference to sustenance).

- **If applicable, is the statistical analysis and its interpretation appropriate?**

Formal statistical analysis is not really relevant. Extrapolating observations made from a very small number of published papers to inferences has many limitations, particularly as all papers originate from a few countries. Papers from India dealing with telehealth and SLP are conspicuous by their absence (refs 10, 11). These need to be discussed and have been elaborated on in the reviewer’s main report.

- **Are all the source data underlying the results available to ensure full reproducibility?**

The extended data and additional information are available as supplemental files.

- **Are the conclusions drawn adequately supported by the results?**

Conclusions drawn can be considered to be reasonably supported so far as it refers to the data from the papers analysed. The reviewer has pointed out that in the real world there are many success stories of implementation of telehealth in LMICs that have not been formally published due to various reasons. The authors would do well to add a paragraph on limitations and even dangers of drawing conclusions going only by published material (even if “if it is not published it does not exist”). Implementing telepractice in SLP is not the same as implementing telepractice in other clinical domains. Discussion on commonalities and dissimilarities would be useful particularly if numbers are furnished from LMICs and other countries to show that virtual remote delivery of SLP is not a luxury but a necessity.

**MINOR:**

- **Title: **'Understanding the implementation of telepractice in speech and language services for children and adults using a mixed-methods approach' - adding the words *children and adults* do not give additional meaning and may be dropped.

Is the work clearly and accurately presented and does it cite the current literature?

Yes

If applicable, is the statistical analysis and its interpretation appropriate?

Not applicable

Are all the source data underlying the results available to ensure full reproducibility?

Yes

Is the study design appropriate and is the work technically sound?

Yes

Are the conclusions drawn adequately supported by the results?

Yes

Are sufficient details of methods and analysis provided to allow replication by others?

Yes

Reviewer Expertise:

Telemedicine, Telehealth, Neurosurgery

I confirm that I have read this submission and believe that I have an appropriate level of expertise to confirm that it is of an acceptable scientific standard.

## References

[1] : Telehealth in India: The Apollo Story and Tele emergencies in the Himalayas. *Presented at the 23rd ISfTeH International Conference eHealth 2018 and Annual Conference of the Finnish Society of Telemedicine and eHealth March 15-17 2018* .

[2] : Technology Enabled Remote Healthcare in Public Private Partnership Mode: A Story from India.2021; 10.1007/978-3-030-56917-4_14 197-233 10.1007/978-3-030-56917-4_14

[3] : Strategy and plan of action on eHealth. *51st Directing Council, 63rd Session of the Regional Committee* .2011; Reference source

[4] : Digital Health Care in Public Private Partnership Mode. *Telemed J E Health* .27(12) : 10.1089/tmj.2020.0499 1363-1371 10.1089/tmj.2020.0499 33819433

[5] : A model for sustainable, partnership-based telehealth services in rural India: An early process evaluation from Tuver village, Gujarat. *PLoS One* .2022;17(1) : 10.1371/journal.pone.0261907 e0261907 10.1371/journal.pone.0261907 35025902PMC8757919

[6] : Designing Cost-Effective Telemedicine Camps for Underprivileged Individuals in Less Developed Countries: A Decomposed Affordance-Effectivity Framework. *Journal of the Association for Information Systems* .2020;21(5) : 10.17705/1jais.00637 1279-1312 10.17705/1jais.00637

[7] : Strategy in the time of pandemics, climate change and the Kurzweil Singularity. *Journal of Strategy and Management* .2021;14(3) : 10.1108/JSMA-07-2021-0146 300-314 10.1108/JSMA-07-2021-0146

[8] Management Science and Practice Deployed in Providing Technology Enabled Remote Health Care. *Lecture given at IIM Ahmedabad India* .2019;

[9] : National Programme for the Prevention and Control of Deafness. Reference source

[10] : Experience Delivering Tele-practice Services among Upcoming and Working Professionals of Speech Language Pathology. *Journal of Health and Allied Sciences NU* .2022; 10.1055/s-0042-1743550 10.1055/s-0042-1743550

[11] : A Survey of Telepractice in Speech-Language Pathology and Audiology in India. *Int J Telerehabil* .2017;9(2) : 10.5195/ijt.2017.6233 69-80 10.5195/ijt.2017.6233 29238451PMC5716619

# Response

**Published:** 2022-05-18

**Authors:** Vidya Ramkumar

**Affiliations:** Sri Ramachandra University Chennai, India

We thank the reviewer for his valuable comments and have provided our responses and clarifications for each of them in the section below. Edits have been made using track changes and existing information pertaining to certain comments by the reviewer has been highlighted in yellow for ease of reference.

**Comment 1: **Discussion regarding sustainability, more information on the business model, cost-effectiveness, some data on the market for telepractice, and some info on the acute shortage of trained audiologists and speech experts would be useful. Some statistics from a few countries in different income brackets ( https://datahelpdesk.worldbank.org/knowledgebase/articles/906519-world-bank-country-and-lending-groups) would convince the reader that this gap in SLP management is unlikely to be redressed without virtually extending the reach of the limited domain experts available.

**Response 1: **We have added information on available literature that describes cost-effectiveness (page 3 - line 15). While there was no data available on the business model, sustainability aspects were captured in this study and have been reported as implementation barriers and facilitators. We agree that information regarding the shortage of trained speech and language pathologists across the world will shed more light on the need and advantages of telepractice. This information has been added in the introduction section of the article (page 3 - line 10).

**Comment 2:** While the primary intent of this study is to summarise the current world literature (choosing articles fulfilling very rigid criteria), it perhaps may not be irrelevant to include interviews with others deploying telepractice in SLP in India, including the authors themselves (assuming that the quotes could be subsumed under Personal Communication). This addendum may add to the perspective.

**Response 2: **While anecdotal evidence suggests that tele-speech and language pathology is in practice in India, there is limited literature available. Even the available literature did not fit the inclusion criteria of being long-term sustained efforts of implementation and therefore none from India could be included in the interview. This information has been mentioned in the discussion section of the article (page 19 - line 18).

**Comment 3:** The low availability and accessibility of SLP services in low and middle-income countries (LMICs) can be discussed. Authors have pointed out that none of the papers studied in deploying telepractice in SLP was from LMICs. It needs to be pointed out that even if such projects were being executed in LMICs, the priority for publishing would be low. Publishing is a time-consuming, labour incentive process, particularly in a non-academic milieu.

**Response 3: **General information regarding the low availability and accessibility of SLP services in LMICs has been mentioned in the introduction section of the article (page 3 - line 8). We agree that the priority and feasibility of publishing in LMICs might be considered low and have added this as a point to consider in the discussion section (page 19 - line 22).

**Comment 4: **Though implied, the authors need to reiterate that their conclusions are entirely a synopsis of the 11 studies shortlisted. Five projects from the USA, two from Australia, and one each from Norway and Canada do not represent the world.

**Response 4: **Although this has been mentioned in the article (page 11 - line 2), we have reiterated the same in the discussion section (page 20 - line 33) to add emphasis to the restricted context of views obtained from the semi-structured interviews.

**Comment 5: **One or two paragraphs highlighting the limitations of the methodology used in this study would add value to the paper.

**Response 5: **We used the standard guidelines recommended for scoping review and semi-structured interviews. However, the quality appraisal could not be performed due to the limited number of studies and has been mentioned on page 20 (line 32). The restricted context of the views obtained from the interview has also been included in the discussion section (page 20 - line 33).

**Comment 6:** Reliable information available in the public domain, though not in indexed journals, could also be mentioned.

**Response 6:** In order to accommodate for non-indexed publications, grey literature was included in the review process by searching databases such as Google Scholar and PubMed, as described in the method section (page 4 - line 33). Therefore, we believe we have considered all reliable information available in the public domain.

**Reviewer’s additional comments**

**MAJOR:**

- **If applicable, is the statistical analysis and its interpretation appropriate?**

Formal statistical analysis is not really relevant. Extrapolating observations made from a very small number of published papers to inferences has many limitations, particularly as all papers originate from a few countries. Papers from India dealing with telehealth and SLP are conspicuous by their absence (refs 10, 11). These need to be discussed and have been elaborated on in the reviewer’s main report.

**Response: **For this study, implementation of telepractice for SLP services was operationally defined as “a sustained model of service delivery using telepractice based on prior feasibility and validation studies”. Any telepractice project implemented for two or more years, providing screening, diagnostic or rehabilitative services to individuals of all age groups with speech and language difficulties across the world, was of interest. Even if the word “implementation” was not used in the articles, words such as “on-going” or the long-term nature of the service implementation described using the number of years were considered.

 While we did not exclude any studies deliberately, the articles mentioned by the reviewer do not fit the study’s inclusion criteria and therefore were excluded.

- **Are the conclusions drawn adequately supported by the results?**

Conclusions drawn can be considered to be reasonably supported so far as it refers to the data from the papers analysed. The reviewer has pointed out that in the real world there are many success stories of implementation of telehealth in LMICs that have not been formally published due to various reasons. The authors would do well to add a paragraph on limitations and even dangers of drawing conclusions going only by published material (even if “if it is not published it does not exist”). Implementing telepractice in SLP is not the same as implementing telepractice in other clinical domains. Discussion on commonalities and dissimilarities would be useful particularly if numbers are furnished from LMICs and other countries to show that virtual remote delivery of SLP is not a luxury but a necessity.

**Response: **Our results are based on scientific publications in journals as well as grey literature such as detailed reports, unpublished dissertations or thesis. We recognise that this will still not capture information that is not available in the public domain or known only anecdotally. We have mentioned this in our discussion section (page 19 - line 22)

**MINOR:**

- **Title: **'Understanding the implementation of telepractice in speech and language services for children and adults using a mixed-methods approach' - adding the words *children and adults* do not give additional meaning and may be dropped.

We agree with the reviewer to modify the title as follows:

 'Understanding the implementation of telepractice in speech and language services using a mixed-methods approach.’ (Page 1 - line 1)
